# Cheryl Cohen: tracking respiratory diseases, informing policy

**DOI:** 10.2471/BLT.20.031220

**Published:** 2020-12-01

## Abstract

Cheryl Cohen talks to Gary Humphreys about how the COVID-19 pandemic is encouraging respiratory disease surveillance collaboration in South Africa.

Q: How did you come to work in your chosen field?

A: I started out as a clinician in South Africa in the late 1990s when the country was struggling with the HIV (human immunodeficiency virus) epidemic. Working in a health system that was regularly overwhelmed brought home to me the limited impact I could have as a doctor and I began to think about the different options open to me. When I was offered a scholarship to study for a master’s in epidemiology at the London School of Tropical Medicine in 2004, I took it.

Q: You felt that you could have a greater impact as an epidemiologist?

A: I felt my work would have an impact on more people, yes. But I was also really drawn to the microbiological side of the discipline. I returned to South Africa from London in 2005 and was hired as an epidemiologist by the NICD which was just getting established at that time. What I love about the job I do now is that it allows me to study the pathogens and the impact they have on human health, but also the broader societal aspects of epidemics in all their complexity.

Q: What is your role at the NICD?

A: I lead the respiratory disease surveillance programmes and I also head up a respiratory diseases research team which looks at burden of disease, risk groups and transmission as well as assessing the impact and effectiveness of different public health interventions. We’re currently doing a lot of work on COVID-19, as you might expect.

Q: South Africa has been particularly hard hit by the COVID-19 pandemic. How has it impacted your work?

A: On a personal level, the early period of the pandemic was the most challenging. The scale and pace of the explosion of cases was more than we had seen before. There was also a lot of pressure from the media and a genuine need to provide real-time, reliable information to the public. All of this led to many late nights and high stress calls and meetings over weekends and in the evenings. So, this period was really difficult for me and other members of my team. On top of everything else, several staff were assessed as high risk and so removed from direct patient contact, which reduced our surveillance capacity. We also had some COVID-19 cases among clinical teams and other members of the team were assessed as close contacts and so had to quarantine. Fortunately, we received financial help from a local fund set up to support COVID response which allowed us to hire additional staff, and other departments within our institute offered to help with surveillance management. In the end our systems remained running throughout.

Q: What is the focus of your COVID-19 work?

A: We have several ongoing studies, including one covering SARS-CoV-2 transmission, and another covering burden of COVID-19 and risk factors. The transmission study – known as PHIRST-C – is designed to establish levels of SARS-CoV-2, and transmission in individual households in rural and peri-urban settings in South Africa. Participants are being tested twice weekly until the end of 2020 using PCR (polymerase chain reaction) tests to detect the presence of viral genetic material, and serological tests to detect the presence of antibodies. Participants are also being asked if they have experienced any COVID-19 symptoms. One of the questions we’d like to answer is how prevalent asymptomatic infection is and the degree to which asymptomatic people transmit the virus compared to those with symptoms. The study is also an opportunity to examine the role played by children in virus transmission.

“We have registered just a couple of cases of influenza.”

Q: One of the key concerns with SARS-CoV-2 is the possibility of reinfection. Is that something you are looking at?

A: Yes. We are going to be able to study reinfection in the PHIRST-C study and will be focused on obtaining viral sequence data from individuals with suspected reinfection. We are also testing for influenza and RSV (respiratory syncytial virus) which causes respiratory tract infections and is a major cause of lower respiratory tract infections and hospitalization of children during infancy and childhood.

Q: What made you decide to study different pathogens at the same time?

A: The PHIRST-C study builds on an earlier multi-pathogen study which ran for three years in two locations in South Africa. So, on the one hand, we were making good use of a surveillance structure that we’d already set up, but there was also a desire to answer questions being put by South African Department of Health officials regarding the possible impact of parallel COVID-19 and flu infections as we moved into our flu season, which runs from around mid-June to around the end of August. Back in April there was a real concern about the threat posed by parallel flu and COVID-19 seasons, notably with respect to possibly overwhelming demand for diagnosis and treatment. The PHIRST-C and burden of disease studies were viewed as opportunities to build the knowledge base required to meet those and other challenges.

Q: Is the burden of disease and risk factor study also a multi-pathogen exercise?

A: No, the study is focused on the burden of disease and risk factors associated with SARS-CoV-2 infection. We are seeking to establish the number of deaths, hospitalizations, mild disease and asymptomatic populations. We are also looking at the disease burden in different populations and different ages. That said, we are building on similar studies conducted for influenza, using the sentinel surveillance sites set up for the ILI (influenza-like illness) and SARI (severe acute respiratory illness) surveillance systems, supplemented with additional health-care utilization surveys. We know that around half of respiratory disease deaths in South Africa occur outside the hospital and we’d like to know what the proportion is for COVID-related deaths.

Q: As I understand it, South Africa has had a very mild flu season. Can you confirm that?

A: Mild would be an understatement. We have registered just a couple of cases of influenza in our transmission study cohort, and our ILI and SARI surveillance systems present a similar picture at the national level, so the data are pretty robust. Those systems have been running since 1985 and in a normal winter register hundreds of thousands of respiratory illness cases per week including influenzas. Since the lockdown which ran from 26 March to 1 May we have picked just a handful of flu cases through ILI and SARI. We also didn't really have an RSV season this year. Again, that is unprecedented.

Q: There have been reports of mild-to-non-existent flu seasons in several Southern Hemisphere countries. What do you think is driving this apparent trend?

A: It’s possible that the various containment measures used to flatten the curve of COVID-19 transmission, including social distancing, school closures, hand washing and mask wearing, are having an impact. Other factors such as the closing of borders and putative virus–virus interactions are alternative explanations.

“COVID-19 has brought people out of their data silos.”

Q: You mentioned the questions put by policy-makers in anticipation of parallel flu and COVID-19 epidemics informing your research choices. How closely do you work with the health ministry?

A: A core function of the NICD is to provide microbiology, virology, epidemiology, surveillance and public health research to support the government’s response to communicable disease threats. So, I work closely with the Department of Health to generate evidence to guide policy with regard to the control and management of respiratory diseases.

Q: Do the policy-makers listen to you?

A: They do. It helps that Dr Zwelini Mkhize, our minister of health, is a doctor and sees the value of science and of the evidence on which science must be based. He has also done a great deal to leverage the power of the institutions needed to develop an effective response. This includes being supportive of our work, but also taking steps such as setting up ministerial advisory committees to get the best information and analysis.

Q: *The politicization of the COVID-19 response has been highlighted in many countries. Has it been a factor in South Africa?*

A: I think the politicization of the COVID-19 response has been a factor everywhere. The need to balance different public health and socioeconomic considerations in a rapidly evolving pandemic and with very limited data makes it inevitable. But even putting the pandemic aside there are always political pressures, and prioritization is not always evidence based. It’s our job to make sure that the best evidence is at least considered.

Q: How do you go about achieving that?

A: Partly by making sure that we are answering questions people want answers to, and in a way that they can understand and use. But I think that it’s more than just a question of generating good actionable intelligence. Informing policy is sometimes represented as an essentially technical process whereby researchers feed information through to the decision-makers. In fact, it’s more interactive than that, and requires an ability to respond to the evolving policy context and to take opportunities as they arise.

Q: What opportunities, if any, are coming to light in the context of the pandemic?

A: COVID-19 is really boosting surveillance collaboration and momentum. There are things we have been wanting to do on respiratory disease surveillance for years that we have never been able to do, but which are now becoming possible. For example, one of the obstacles we have always faced in trying to set up real-time surveillance of deaths is access to good, timely mortality data. As a result of collaborations brought about in response to the COVID-19 pandemic we are now working together with the Medical Research Council who have access to these data. Also, players in the private health insurance industry are now making their information available in reports. COVID-19 has brought people out of their data silos. There has also been a significant increase in funding from donors to support various aspects of COVID-19-related surveillance and research, but the increased willingness to share data and to collaborate is for me the real silver lining of this terrible pandemic. I hope that collaboration will continue once it is all over.

